# Personal travel blogs as texts for studying intercultural interactions: a pilot test case study of an American sojourner’s blogs from Zimbabwe

**DOI:** 10.1186/2193-1801-3-211

**Published:** 2014-04-28

**Authors:** Rick Malleus, Elizabeth Slattery

**Affiliations:** Communication Department, Seattle University, 304 Lynn Hall, 901 12th Avenue, Seattle, WA 98122 USA; English Department, Indiana University East, 2324 Chester Boulevard, Richmond, IN 47374 USA

**Keywords:** Travel blogs, Culture shock, Zimbabwe, Cross-cultural comparison

## Abstract

This paper makes the argument that personal travel blogs are an important site for studying self-reports of face-to-face intercultural interaction. The guiding research question is “Are personal travel blogs good sources of intercultural communication data?” A content analysis of an American woman’s travel blog, written on a sojourn to Zimbabwe, was performed using four intercultural communication constructs that served as frameworks for developing a rubric and for analysis. Those constructs are: culture shock, intercultural communication challenges, cross-cultural comparison and intercultural adaptation. Results provide evidence of written reflections by the blogger in all four coding categories. The evidence of culture shock provided in the blog was multifold, multifaceted, and congruent with many of the well-established elements of culture shock reported in the field. The evidence of cross-cultural comparison in the personal travel blog was, overwhelmingly, comprised of reflections comparing host and home cultures, both in environment and cultural practices. There was limited evidence of reflections about communication challenges or adaptation by the blogger on her sojourn. The article concludes with a discussion of the implications these findings have for the potential travel blogs might provide for analysis of intercultural communication as well as addressing the limitations of the study’s findings.

## Introduction

This paper makes the argument that personal travel web logs (blogs) are an important site for studying self-reports of intercultural interaction in a globalized world that are not being utilized by intercultural communication scholars. “Are personal travel blogs good sources of intercultural communication data?” is the research question guiding the study. Shuter (2012) calls for “lines of research on how ICT’s affect intercultural communication between individuals and groups” that challenge the “more than 50 years of intercultural communication knowledge and theory rooted in twentieth-century paradigm of face-to-face interaction” (p. 220). This study, however, tests the idea of using ICTs as a channel for studying blogs and analyzing bloggers’ reflections on the face-to-face intercultural interaction they are engaged in while traveling in a host culture. This research approach suggests the importance of studying face-to-face intercultural interaction, but also recognizes the important role the Internet plays in providing intercultural communication scholars texts to study.

An important part of the globalization process is the interconnectedness of information that is available to people and the ability people have to communicate with each other through that interconnected technology Kim and Bhawuk (2008). Blogs are a platform that allow for both the global dispersion of information, and for communication between bloggers and their audience. Blogs can “serve as thriving sites of intercultural communicative exchange when they are unmediated—or, at least, minimally mediated—by third parties” (Pfister and Soliz 2011, p. 247). This relatively unmediated communication that blogs provide have the potential to allow for intercultural communication to take place, but are also platforms for bloggers to reflect on their own face-to-face intercultural experiences. Globalization has offered new culture learning opportunities Kim and Bhawuk (2008) and this study seeks to examine if blogs might provide those opportunities for studying reflections on intercultural interactions.

In this study, intercultural communication is defined as “a transactional, symbolic process involving the attribution of meaning between people from different cultures” (Gudykunst and Kim 2003, p. 17). As Gudykunst and Kim (2003) suggest, the communication does not have to be effective to be considered intercultural communication. Interpersonal, face-to-face interactions where people from “different cultures are trying to share ideas, information and feelings” (Samovar and Porter 2003, p. 2) are the focus of intercultural communication interest. This study also assumes that contexts (cultural, environmental, perceptual and socio relational) are key in understanding intercultural communication (Neuliep 2012; Samovar and Porter 2003). Finally, intercultural communication is conceptualized as a process that can challenge communicator’s assumptions about the world, can help broaden understanding and provide windows on other ways to live that challenge “taken-for-granted beliefs” (Sorrells 2013, p. 15).

The objectives of this paper are fourfold. The paper argues that blogs are a rich set of texts ripe for studying reports of real-world intercultural interactions that are not currently being studied by intercultural scholars, and it demonstrates the cogency of this argument by presenting a case study that analyzes personal blog posts of an American sojourner in Zimbabwe. This paper also considers both the benefits and limitations on intercultural communication research of studying personal travel blogs as reports of intercultural interaction.

## Literature review

### Blogs and bloggers

New media are “transforming communication across cultures” (Shuter 2012, p. 219). Among those new media enabling communication across boundaries of geography and time are blogs Shuter (2012). Blogs are growing in visibility (Lee and Gretzel 2014; Whalen et al. 2013; Burgess 2006) and have characteristics which make them popular including: the low barriers there are to the creation and maintenance of a blog, their ease of use, relatively easy interactivity and a blog’s potential for being widely distributed Reese et al. (2007). In addition, blogs serve as places for exchanging information between bloggers and their audience Allan (2006). Blogs exemplify the claim that Pfister and Soliz (2011) make about internetworked communication, that gatekeepers cannot “patrol the boundaries of what shall and shall not be made public by people from other cultures” (p.247).

Cultural norms play a role in the way people form perceptions of, and how they interpret, blog content about countries and cultures other than their own Sun et al. (2014). Culture has also been found to play a role in how blogs are used Lee and Gretzel (2014). Research has found travelers who visited other cultures and who kept blogs tended to focus on four themes Snee (2013). Those themes were the exoticness of the places they visited, a feeling of being out of place in their host cultures, interactions with host culture nationals and construction of place as different, tied to historical legacies Snee (2013). Some audiences have found personal blogs to be credible sources with audience characteristics and motivations for reading blogs being important to consider Kaye and Johnson (2011).

The “objectives of bloggers are as diverse as the people who write them” (Gregg 2006, p.154) with different bloggers having different agendas and motivations for maintaining their blogs (Whalen et al. 2013; Burgess 2006). Bloggers can take on the roles of “investigator and provocateur” (Gregg 2006, p. 153) in their writings. Bloggers can also play the role of citizen journalists, functioning as publisher, creator of online content and distributor of that content (Bowman and Willis 2003; Katz and Lai 2009). In addition, Burgess (2006) argues that blogs are a form of cultural production and a place for self-representation. Blogs are a platform where people write about their own life experiences, providing a forum for self-expression and an outlet to create (Hayton 2009; Hollenbaugh 2011). Travel bloggers have multiple reasons for sharing their thoughts with an audience that include how useful their information might be, enhancing reputation, building trust and altruism (Ting et al. 2014). While personal bloggers have been found to report helping, passing time, exhibitionism, social-connection, and archiving to be among the motives for blogging Hollenbaugh (2011).

What qualities do blogs generally possess that might make them appropriate texts for content analysis? Blogs are generally associated with a single author, and allow an archival view into the blogger’s world (Lee 2010; Gregg 2006 Dos and Demir 2013). This archive of information in blogs is found in reverse chronological order and allows a picture of the blogger’s online world to develop Lee (2010). Blogs therefore can be thought of as providing views into spaces and worlds that were previously unavailable Laff (2007). Here the idea of bloggers having an audience is important to remember, as research has demonstrated that having an audience to share those views with enhances the blogger’s writing Lee (2010).

Another characteristic is that blogs can be both a communal space and/or can be a private space (Lee 2010) and generally have an intentional quality to them Gregg (2006). Bloggers often use examples from their own lives in their posts, so it can be argued that blogs are constructivist in nature Dos and Demir (2013). Further, this characteristic of blogs suggests that people represent themselves and their experiences rather than simply being represented by others Pfister and Soliz (2011). This idea has important implications for intercultural communication as multiple perspectives can be seen online Pfister and Soliz (2011) and being able exposed to multiple views, different ways of doing and seeing the world are important way to build intercultural skills and understanding. Freeman and Brett (2012) position that real-world bloggers’ writing is timely, frequent, and interest-driven are important qualities to consider when thinking about blog posts as potential texts for analysis. These qualities suggest that blogs allow you to think aloud, with bloggers able to post whenever they feel moved to write Chong (2010) enabling “critical refinement and thinking-in-process” (Gregg 2006, p. 154).

Perhaps the most important characteristic that many blogs have that would make them suitable texts for intercultural communication research may be the idea that blogs can be reflective in nature. Blogs can be channels for reflection and the blogging process is seen as a way to enhance and encourage reflective thinking (Chong 2010; Lee 2010; Bonk and Zhang 2006; Dos and Demir 2013; Gregg 2006). The medium encourages self-expression and potentially interactive exchange, allowing critical reflection with bloggers writing at their own pace Lee (2010). As Freeman and Brett (2012) assert, bloggers’ posts are a personal “interpretation of their experiences, thereby revealing their interests and providing frequent insight into the communities in which they participate” (p. 1033). Those insights are often arrived at through the reflective writing process.

### Reflective writing

Watton et al. (2001), define reflective writing as “a means of turning ‘surface’ learning into ‘deep’ learning.” They posit that reflective writing is not simply “descriptive” writing, but that it explores the motives of both the writer and of others while investigating how behaviors and reactions are related. They further assert that reflective writing deeply questions ideas while requiring an objective consideration of other perspectives than the writer’s own, in addition to finding connections in ideas, events, and thoughts. Finally, they note that reflective writing acknowledges that the writer’s point of view can alter because of the passage of time in both his or her own emotions at the moment of the writing and also because of reviewing said external information.

According to Lee (2010) blogs offer an opportunity for critical reflective writing because of both their self-expressive and social networking qualities. Similarly, Dos & Demir (2013) assert that blogs can amplify reflection, depth of learning, and knowledge bases. Though not all blogs are reflective in nature, the focus of this article is on those that demonstrate similar qualities of reflective writing as presented above. It is this depth of critical reflection that can provide rich, specific detail about intercultural interactions to be revealed in blog posts about travel. Freeman and Brett (2012) determined three types of reflection that are crucial to such blogging. These include *descriptive reflection*, which attempts to provide reasons for actions taken, the blogger’s personal experience or purpose of the blog; *dialogic reflection*, which sees the blogger stepping back from the subjective to the more objective perspective, wherein he or she identifies inconsistencies, differing points of view, multiple solutions, etc.; and *critical reflection*, wherein the blogger looks externally at multiple historical, socio-political, cultural, or ethical influences/results.

In an exploration of student teacher perspectives in reflective writing, Bain et al. (2002), applied their “5Rs” framework to explain the different levels of reflection. These include Reporting, Responding, Relating, Reasoning, and Reconstructing. This framework indicates that the complexity of the writing (and indeed of thought) increases as the writer moves through the levels, beginning with the simplest act of recording an event/description and building towards a product that includes an application of some critical thought. Though the criteria establishing what makes a piece of writing (in this instance, a blog) “reflective” according to these scholars differ slightly, the similarities amongst them include deepening understanding of the self and the broader world by relying on both subjective and objective information.

### Intercultural communication frames

In addition to the discussion of blogs and reflective writing above, it is necessary to briefly discuss four intercultural communication theoretical frames in this section of the paper. These intercultural constructs serve as frameworks for developing a rubric and for analysis. Those constructs are: culture shock, intercultural communication challenges, cross-cultural comparison and intercultural adaptation.

### Culture shock

Culture has been defined in many different ways (Gudykunst and Kim 2003; Sorrells 2013). The way this study understands culture is as being “central to the way we view, experience, and engage with all aspects of our lives and the world around us” (Sorrells 2013, p. 3). Culture and communication are highly interconnected (Hall 1959 and Birdwhistell 1970 as cited in Bowman and Willis 2003). When people from one culture move to a host culture and communicate with people from that host culture, they can be surprised, stressed, anxious and can question their identity Gudykunst and Kim (2003) as their communication and ideas about culture meet the host culture. Oberg (1960) is credited with coining the term *culture shock* and there are many different definitions of the term. Watton et al. (2001) effectively summarize important ideas common to many of those definitions: People feel strain as they go through the effort of making all the adaptations necessary in the host culture, people experience a sense of loss as they feel deprived of various things like their friends, possessions and a clear status in society, people feel a sense of rejection or might themselves reject others in the host culture, people experience confusion about role expectations, people feel anxiety, surprise and sometimes disgust as they become aware of cultural differences between their culture and a host culture, and people feel helpless as they cannot cope in the host culture. Further, Furnham (2010) suggests agreement in the field that culture shock is understood as including the idea of “losing the power of easy communication” (p. 87–88) and that this loss “can disrupt self-identity, world views and indeed all systems of acting, feeling and thinking” (p. 88). From a review of the literature, Zhou et al. (2008) found there is some agreement that when people are exposed to new cultures, their responses can be grouped into the affective, behavioral and cognitive domains. In other words, the way sojourners think, feel and act are impacted upon by the host culture. This makes sense as sojourners in a host culture come into contact with “new values, new practices and ways of living” (Xia 2009, p. 97). Therefore, much of the current thinking about culture shock frames the transition as “contact-induced stress accompanied by skill deficits that can be managed and ameliorated, and terms such as ‘adaptation’ and ‘acculturation’ have been increasingly used instead” (Zhou et al. 2008, p. 65).

When considering patterns in the literature about culture shock and student adjustment while in transition to host cultures, Furnham (2010) suggests that “various concepts have been put forward to predict the quality, quantity and chronicity of sojourner distress” (p. 91). Among those identified patterns are culture-distance, which suggests that the degree of difference between home and host culture are related to the amount of “stress or difficulty experienced” (Furnham 2010, p. 91) by the sojourner, and the friendship social support concept that suggests having a network of friends helps sojourners cope with the transition more effectively Furnham (2010). In culture learning models, it is suggested that the ‘shock’ serves as “the stimulus for acquisition of culture-specific skills that are required to engage in new social interactions” (Zhou et al. 2008, p. 65). In stress coping models the ‘shock’ “stems from inherently stressful life changes, so people engaging in cross-cultural encounters need to be resilient, adapt, and develop coping strategies and tactics. Adjustment is regarded as an active process of managing stress at different systemic levels – both individual and situational” (Zhou et al. 2008 p. 65).

Abarbanel (2009) suggests that it is helpful to consider the *signals* as opposed to the *symptoms* of culture shock (a term/frame she does not like to use). Some of those signals are “homesickness, boredom, withdrawal, need for excessive amounts of sleep, compulsive eating or drinking, stereotyping local people, reduced ability to work effectively and physical ailments” (Abarbanel 2009, p. 136). Clearly, there is a range of signs to signal that a sojourner might be going through culture shock. Some of these signs might be expected, like feeling homesick when away from home, but other signs might not immediately be associated with being in a host culture and experiencing culture shock, like excessive eating or drinking. In seeking explanations for behavior, knowing signs of culture shock are useful. It is important however, to recognize that the process of moving from one culture to another differs from person to person, and from culture to culture, and as such, theoretical ideas of adaptation, of which culture shock is a part, should be considered guides to understanding people’s experiences, but that looking at particular cases are also useful to understand experiences like culture shock Sorrells (2013).

### Intercultural communication challenges

Communication links sojourners to their new environment Gudykunst and Kim (2003). Communication challenges that sojourners may face when they cross cultures play a role in culture shock and are worthy of special consideration. This special consideration is due because intercultural interaction can pose both verbal and nonverbal challenges to sojourners as they encounter cultural variability in communication rules and behaviors (Gudykunst and Kim 2003; Neuliep 2012). Two types of miscommunication may occur according to Gass and Veronis (1991): misunderstanding and incomplete understanding. Misunderstandings are characterized by neither party realizing that a problem has occurred during a communicative interaction, while incomplete understanding occurs when at least one person recognizes some communication problem during an interaction.

Part of being an effective intercultural communicator is having message skills. These skills can be thought of as “the ability to understand and use language and feedback” (Jandt 2013, p.36). Four different verbal communication styles reflecting patterns of culture have been identified in the field: direct–indirect, elaborate-succinct, personal-contextual and instrumental-affective Gudykunst and Ting-Toomey (1988). These different verbal styles pose multiple and different communication challenges for sojourners depending on their host culture’s preferences and how those agree or disagree with the sojourner’s home culture preferences. Additionally, it is important to remember that language can mark a person as belonging to an ingroup or being part of the outgroup as language is central to identity (Neuliep 2012 Jandt 2013; Lustig and Koester 2013). Vocabulary and idiomatic equivalence can also be issues in intercultural communication related to language differences Jandt (2013).

In addition to verbal communication challenges, nonverbal misinterpretations or not recognizing nonverbal communication cues have been identified as barriers in intercultural communication (Jandt 2013; Lustig and Koester 2013). Misunderstandings due to cross-cultural differences in nonverbal behavior are in fact fairly common Argyle (1988). Sojourners may expect differences in verbal communication in a host culture, but they often do not expect differences in nonverbal communication Albert and Ha (2004).

Nonverbal communication can be intentional or unintentional, conscious or unconscious, include non-spoken symbolic communication, elements of the environment and can serve multiple functions (Jandt 2013; Lustig and Koester 2013). Nonverbal communication may at times be more trusted than verbal communication. This is especially the case if there is a conflict between the verbal and nonverbal messages that are being sent Ting-Toomey (1999). Sojourners in a host culture may have difficulties making these distinctions in decoding nonverbal behavior. Sojourners in a host culture also might make attributions about nonverbal behavior based on their home cultures and it is likely that different cultural groups will give different interpretations for the same nonverbal behavior” (Albert and Ha 2004, p. 254).

Communication grounded framing of intercultural competence tends to be focused on skill development Jandt (2013). A sojourner’s cognitive, affective and behavioral communication skills and abilities can be thought of as their communicative competence in the host culture and varying levels of competence allow for varying levels of participation in interpersonal and mass communication in the host culture Gudykunst and Kim (2003). In addition, for a sojourner to achieve intercultural competence they need adequate knowledge and a satisfactory level of motivation Lustig and Koester (2013). As Sorrells (2013) suggests, having the knowledge, attitude and skills that are needed to “engage effectively in intercultural situations” (p. 231) can be thought of an intercultural competence. Being motivated to learn about others and understanding how as a sojourner you are positioned in relation to those in the host culture are important elements of this intercultural competence Sorrells (2013).

Host communication competence can be thought of then as “the degree to which the newcomer can encode and decode verbal and nonverbal messages within the host environment” (Neuliep 2012, p.408). Gaining communication competence about ways of communicating in a host culture includes “a refinement of insights and predictions about host behavior. The individual tends to enter the host culture with simple, gross stereotypes that are fine-tuned through face- to-face or mediated interaction” (Reece and Palmgreen 2000, p. 809). One way a sojourner may try to develop their communication competence in trying to understand the host culture is by making comparisons to that with which the sojourner knows and is familiar.

### Cross-cultural comparison

By comparing and contrasting cultural patterns from different cultures sojourners can think about the implications those differences and similarities have for interpersonal communication Lustig and Koester (2013). The word cross-cultural implies “a comparison of some phenomena across cultures” (Gudykunst and Kim 2003, p. 18) with the goal being to perform “a series of intracultural analyses in order to compare one culture with another on the attributes of interest” (Lustig and Koester 2013, p.51). Of course, sojourners do not typically conduct systematic cross-cultural research in trying to work out how a host culture functions, but making cross-cultural comparisons are a strategy often used by sojourners as they try to make sense of their host culture. This is because making cross-cultural comparisons tend to be quite useful in helping sojourners understand cultural differences Lustig and Koester (2013).

Using culture-general information about a culture is one comparative tool intercultural communicators use to make sense of cultural practices Lustig and Koester (2013). Using culture-specific information is another tool that sojourners can use to help understand a particular culture Lustig and Koester (2013). Using this category of information may help reduce uncertainty about behavior in the host culture.

It can be argued that reduction in uncertainty is an important reason for making cross-cultural comparisons. It has been “found that the more we perceive another as similar to ourselves, the more we are about to reduce uncertainty about the person and to form accurate categories of him or her” (Neuliep 2012, p. 327). “Tolerance for ambiguity concerns a person’s responses to new, uncertain, and unpredictable intercultural encounters” (Lustig and Koester 2013, p.71) and by comparing and contrasting host and home country behaviors in specific situations, sojourners may be able to prepare themselves for higher levels of ambiguity than they are ordinarily comfortable with.

### Intercultural adaptation

Over time while engaging in intercultural communication in a host culture there may be changes in a sojourner’s cultural identity Reece and Palmgreen (2000). Part of the reason these changes may come about is that the sojourner engages in the process of cross-cultural comparison and in the building of a knowledge store about the host culture Reece and Palmgreen (2000). Adaptation is a process that involves acculturation (new host culture learning) but it also involves deculturation (suspending and unlearning home culture) as explained as part of Kim’s theory of intercultural transformation Gudykunst and Kim (2003). Adaptation should be thought of as an outcome of acculturation Berry and Sam (1997). Adaptation can be defined as “a process by which individuals upon relocating into an unfamiliar cultural environment, establish (or reestablish) and maintain a relatively stable, reciprocal, and functional relationship with the environment” (Kim 2008, p. 260).

The process of acculturation is complex, includes many factors and involves behavioral and psychological change that takes place as a result of people from different cultures being in contact with each other (Sam and Berry 1995 as cited in Reece and Palmgreen 2000). Both the sociocultural and psychological adaptation of a sojourner are dependent on the degree to which the sojourner maintains home and host culture’s values Ward and Kennedy (1994). Gudykunst and Kim (2003) suggest that communication lies at “the heart of the adaptation process” (p. 373). When sojourners enter a new host culture, their cultural identities may be marked by quite rigid cultural boundaries, but as sojourners adapt over time, these boundaries may become less rigid Reece and Palmgreen (2000). As Kim (1988) suggests, sojourners experience stress in host cultures and they adapt to help reduce their stress, with this stress-adaptation-growth process facilitated by interactions with those in the host culture.

Some sojourners travel between multiple cultures frequently and have meaningful host culture interactions during those sojourns in which acculturation and deculturation take place Kim (2008). These sojourners might undergo an intercultural evolution, achieving a global understanding that allows a universalized perspective Kim (2008). Gudykunst and Kim (2003) suggest that a sojourner who has experienced this adaptive change might have achieved an intercultural communication competence that would allow them a third-culture perspective with perspectives and levels of awareness that transcend one or two particular cultures.

## Method

Having reviewed the relevant literature above to contextualize this research and provide a framework for analysis, the following section lays out the methodology used. This study reports a pilot test to determine the usefulness blogged reflections of face-to-face intercultural interactions might have for intercultural communication scholars. First, the research questions are presented, followed by both a description of the text on which the content analysis was performed and the instrument, a rubric, which was applied to the text. Finally, there is an explanation of the procedure used to apply this rubric.

## Research questions

The study asks the overarching research question “Are personal travel blogs good sources of intercultural communication data?” To answer that question, four specific research questions were posed:

*R1: What evidence of culture shock does a personal travel blog provide intercultural communication researchers?*

*R2: What evidence of cross-cultural comparison does a personal travel blog provide intercultural communication researchers?*

*R3: What evidence of intercultural communication challenges does a personal travel blog provide intercultural communication researchers?*

*R4: What evidence of intercultural adaptation does a personal travel blog provide intercultural communication researchers?*

Each of the questions helped guide the study. They relate to the literature as being important elements in a sojourner’s experiences in a host culture as they interact with host nationals. The questions also provide specific elements around which to construct an analytical rubric that can be applied to a sojourner’s personal travel blog.

## Text

A personal travel blog of a married, forty-three year old American female who traveled to Zimbabwe for a two-week vacation was used as a case study for analysis. The blogger had never traveled to Zimbabwe before, and had posted consistently to her blog during her sojourn. Twelve blog posts of varying length were written on the Zimbabwean sojourn and the blog was publically available to a global audience, but was primarily written for friends and family to read while she was in Zimbabwe. The platform *Blogger* was used to host the blog and this platform was accessible to the blogger while in Zimbabwe.

The blogger volunteered (after the fact) to have her blog serve as text for this pilot test, and to take part in the study as a member of the research team, hence the choice of her blog for this pilot study. The blog posts translated to 16 typed pages of single-spaced text, with the longest post being 1821 words and the shortest post being 99 words long. The 12 blog posts served as the text for content analysis. (Note: Reader comments on the blog were not included in this study as they were outside the scope of interest for the study, but are important texts to consider in further studies.)

## Instrument

To analyze the content of the blog posts, a rubric was developed, prior to reading the blog. It was developed prior to the reading, not afterwards, in order to avoid bias in the construction of the instrument. The rubric included elements that might be expected to be included in the written reflections of a sojourner experiencing a new culture for the first time. The rubric included four elements: Culture Shock, Intercultural Communication Challenges (verbal and nonverbal), Cross-Cultural Comparison, and Intercultural Adaptation. Each of those elements of the rubric was operationalized in detail based on the relevant literature discussed earlier. The rubric is as follows:

Culture Shock

- A sense of the loss of common cues that tell a person how to behave and how to communicate appropriately. Sense of disorientation.
- Loss of ability to accurately make sense of situations and environments due to cultural differences.
- Losing the power of easy communication.
- Feeling deprived of things a person is used to like friends, family, possessions, food, drink, ways of living etc.
- A sense of confusion about role expectations (for self and for others).
- Feeling helpless and/or not able to cope normally in different situations.
- Feeling surprised, anxious, worried, stressed by situations encountered in the host culture.
- Questioning identity (personal and cultural).
- Feeling homesick.
- Feeling bored.
- Withdrawal.
- Negative stereotyping of host culture and people.

Cross-Cultural Comparison

- Making comparisons between home and host cultures in ways of thinking, behaving, values, communication etc.
- Making comparison between host and another culture in ways of thinking, behaving, values, communication etc.
- Making comparisons between home and host culture environment.
- Making comparisons between host and another culture’s environment.

Communication Challenge

- Expressed difficulty understanding verbal communication in the host culture.
- Expressed difficulty making self understood verbally in the host culture.
- Expressed difficulty understanding nonverbal communication in the host culture.
- Expressed difficulty in knowing how to communicate nonverbally in the host culture.

Intercultural Adaptation

- Demonstrating the use of coping strategies or tactics that are appropriate in the host cultural context.
- Using language/terms from host culture.
- Being able to explain different behavior in clear terms demonstrating understanding of cultural differences.
- Attempting/engaging in newly learned culturally appropriate behavior in the host culture.

## Procedure

Two independent coders (not the authors) were trained and provided with a set of coding instructions and the rubric described above. The coders then independently applied the rubric to the text of twelve blog posts. Coders were asked to clearly mark and label sentences in the text that provided evidence of the four different coding categories. Text could be coded as belonging to more than one coding category. Only text that was coded *by both coders* as fitting into a coding category was included in the results; a measure to ensure reliability in the coding of data.

## Results

Evidence was found in the text of reflections by the blogger in all four coding categories. These were culture shock, communication challenges, cross-cultural comparison and intercultural adaptation. A total of 88 sentences/paragraphs from the blog were coded as belonging to a coding category and were distributed as shown in the table below (Table 1).

**Table 1:** **Summary of coding categories**

Coding categories	Number of instances
Culture shock	25
Cross-cultural comparison	42
Communication challenges	6
Intercultural adaptation	15

Blog reflections on culture shock (28%) and cross-cultural comparison (48%) were found with higher frequency than those on communication challenges (7%) and intercultural adaptation (17%). Textual examples did not always fall neatly into one rubric category only and were therefore coded as evidence of multiple categories. The following example was coded as culture shock and cross-cultural comparison: “At home, a house without screens or visible windows would only be seen in a poor neighborhood, for instance, so when I see this in Rick’s neighborhood, my brain gets confused.” The blogger exhibited an element of culture shock as she was not able to make sense of the environment, as she would be able to at home. At the same time she was engaging in a cross-cultural comparison of what a poor neighborhood in the U.S.A. would look like versus the neighborhood in Zimbabwe that she was not able to easily categorize.

What evidence of culture shock does a personal travel blog provide intercultural communication researchers? The table below shows the distribution of blog posts coded as belonging to a culture shock category (Table 2).

**Table 2:** **Culture shock**

Coding categories	Number of instances
A sense of loss of common cues that tell a person how to behave and how to communicate appropriately.	1
Loss of ability to accurately make sense of situations and environments due to cultural differences. Sense of disorientation.	5
Losing the power of easy communication.	1
Feeling deprived of things a person is used to like friends, family, possessions, food, drink, ways of living, etc.	3
A sense of confusion about role expectations (for self and for others)	0
Feeling helpless and/or not able to cope normally in different situations.	5
Feeling surprised, anxious, worried, or stressed by situations encountered in the host culture.	5
Questioning identity (personal and cultural)	1
Feeling homesick.	1
Feeling bored.	0
Withdrawal	1
Negative stereotyping of host culture and people	2

Three out of the twelve culture shock coding elements were mentioned in posts more often than others, representing 60% of the reflection on culture shock found in the blog. The sojourner’s blog offers several examples that demonstrate different aspects of culture shock. The most prevalent are in the areas of disorientation, helplessness, and anxiety produced in the host culture. Three examples are provided below to illustrate.

The blogger discusses her disorientation and inability to accurately make sense of a holiday she is accustomed to celebrating within the context of a certain (colder) climate: “It didn’t feel much like Christmas to me at all but I know it did for Rick & I’m glad for that. I think I just can’t wrap my brain around ‘summer’ and ‘Christmas.’ I know that is silly--that people all over the world and in California etc. have lovely warm Christmasses, but I kept thinking, ‘How is this Christmas?’ The cultural cues from home that tell her it is Christmas time were missing in Zimbabwe and she found this disorienting.

In a second instance, she reports feelings of both anxiety and helplessness because of regular power cuts. In the blog, she reports, “The [back-up] lights remind me of nightlights and give off a shadowy sort of half light, and I can see how it’s one hundred times better than pre-inverter days, but it makes me feel depressed and a little panicky because I do not trust that the lights will ever come back on again. . . .” Lacking a regular supply of electricity in Zimbabwe contributed to the blogger feeling that she lacked control over her environment, contributing to her culture shock.

Though tongue-in-cheek, the blogger indicates surprise and a certain amount of worry that a trip to a game park is different than she imagined. Her preconceived idea of an area for protected wild game is actually one used for hunting. She says, “I was a bit disturbed to learn that ‘reserve’ is a loose term as there is also some hunting there and now I have visions of just getting a glimpse of some glorious African animal I’ve only ever seen on *Mutual of Omaha’s Wild Kingdom*, only to have it gunned down in front of me.” The blogger had definitions of specific places, like a game park, in mind that were based on her own culture and it was surprising when those definitions of place were challenged in the Zimbabwean host culture.

What evidence of cross-cultural comparison does a personal travel blog provide intercultural communication researchers? The table below shows the distribution of blog posts coded as belonging to a cross-cultural comparison category (Table 3).

**Table 3:** **Cross-cultural comparison**

Coding categories	Number of instances
Making comparisons between home and host culture’s ways of thinking, behaving, valuing, communicating, etc.	17
Making comparisons between host and another culture’s ways of thinking, behaving, valuing, communicating, etc.	1
Making comparisons between home and host culture’s environment.	18
Making comparison between host and other cultures environment.	6

Blog posts representing reflections on cross-cultural comparisons between the home and host culture by the sojourner accounted for 83% of the coded data for this element. Cross-cultural comparisons of host/home environment and of host/home culture were found to be almost equally present in the blog. The blog also offers multiple examples of cross-cultural comparison, particularly in areas that have to do with comparing both the behaviors and environment between the host culture and her home. The following example illustrates differences between what the blogger is familiar with both in terms of the environment but also in terms of how people behave and what they value:

And yes, I was feeling like a lazy American. (Rick might well ask, “How is this different than the days when you sleep and I do the laundry?” and I have no answer, but it is different. I don’t know Eunice and she does not get the benefit of wit and halfway decent backrubs that make up for my domestic failures when Rick and I are in Seattle. I'm still working through my own middle-class American sense of this, but will be very grateful to go to the game reserve tomorrow with freshly laundered and pressed clothes.

Here the blogger reflects on her own middle class values and how those values seem out of place in Zimbabwe, not as easily “fitted in” to what she was experiencing, having a domestic worker doing her laundry for her which made her feel uncomfortable. Yet, at the same time, she was grateful for the services the worker was providing.

The following is an example where the blogger compares the host and another culture’s environment, in this case, Ireland:

I was imagining us all crammed into a stuffy, pea-sized Internet shop like I’m used to in Ireland, but instead, we went to this beautiful cafe with gift shop and sat on the verandah overlooking a shady courtyard with fountains. Sitting there I got some sense (not confirmed, so keep in mind this is just a guess) of what Zimbabwe looked like before things got difficult.

Here, the cross-cultural comparison the blogger makes is regarding expectations. In this instance, her expectations around what an Internet café would be like are based on her previous intercultural sojourn in Ireland and are inaccurate. The Zimbabwean Internet café is big, beautiful and tranquil, in direct contrast to the small, crowded café in Ireland.

What evidence of intercultural communication challenges does a personal travel blog provide intercultural communication researchers? The table below shows the distribution of blog posts coded as belonging to a communication challenges category (Table 4).

**Table 4:** **Communication challenges**

Coding categories	Number of instances
Expressed difficulty understanding verbal communication in host culture.	1
Expressed difficulty making self understood verbally in the host culture.	3
Expressed difficulty understanding nonverbal communication in the host culture.	2
Expressed difficulty in knowing how to communicate nonverbally in the host culture.	0

Reflections on communication challenges experienced by the sojourner in the host culture represented only 7% of the total coded categories in the blog. This was the least reflected upon element in the blog. The blogger records concern about making herself understood in the following example:

Rick had asked me to be sure and tell Eunice how beautiful the house looked and what a wonderful job she’d done preparing our room. (It was spotless, cozy, and I shudder to think what she’d make of our often dusty and always messy Seattle apartment). I did, but was painfully inadequate at expressing myself and instead felt like a well-behaved six- year-old, parroting what her parents have told her to say for politeness sake.

In this example, the blogger reflects on the difficulties she felt in enacting the expected communicative behaviors in Zimbabwean culture where it would be culturally appropriate for her to comment on the good job the domestic worker had done preparing the house she was staying at in the host culture. The experience of both having a house and room prepared for her by someone she would be interacting with daily and having to communicate with that person directly was contrary to her previous cultural experiences, and hence she reports that the communication seemed awkward and unnatural.

What evidence of intercultural adaptation does a personal travel blog provide intercultural communication researchers? The table below shows the distribution of blog posts coded as belonging to an intercultural adaptation category (Table 5).

**Table 5:** **Intercultural adaptation**

Coding categories	Number of instances
Demonstrating the use of coping strategies or tactics that are appropriate in the host cultural context.	2
Using language/terms from host culture.	6
Being able to explain different behavior in clear terms demonstrating understanding of cultural differences.	4
Attempting/engaging in newly learned culturally appropriate behavior in host culture.	3

Reflections on intercultural adaptation demonstrated by the sojourner in the host culture represented 17% of the total coded categories in the blog. This was the second least reflected upon intercultural communication element in the blog. Using language/terms from the host culture represented 40% of the intercultural adaptation seen in the blog, demonstrating the blogger’s use of language and terms from the host culture, such as the following example: “Last night, Mike, the braai-master, cooked chicken, pork belly, and boerevors (a kind of sausage). Edward, the cook (you can have catered holidays there), made us sadza, a staple food here that is made from corn. (It reminded me of rice or mashed potatoes, though the Malleui like it with a tomato gravy on it.” In this excerpt, the blogger is using terms like *braai* (barbeque), *sadza* (maize meal porridge) and *boerevors* (akin to a bratwurst) to appropriately identify elements of objective culture in the same way a Zimbabwean might identify those things. Doing this illustrates the blogger’s language adaptation and use of the appropriate words to label things in her host culture.

## Discussion

The results from this pilot case study demonstrated the potential travel blogs have of providing intercultural researchers with data for analysis about face-to-face intercultural interactions. In four important areas of intercultural communication (culture shock, communication challenges, cross-cultural comparison and intercultural adaptation) the blog provided data for analysis. So the answer to the overarching research question “Are personal travel blogs good sources of intercultural communication data?” appears to be “yes.”

The archival nature of the blog lent itself to some continuity in reflections by the blogger on all four elements of intercultural interaction being studied. The coded posts in this blog showed evidence of being written in a timely fashion (as events unfolded), were interest driven and at times demonstrated writing as ‘thinking-in-process’—all consistent with the literature (Freeman and Brett 2012; Lee 2010; Dos and Demir 2013 Chong 2010; Gregg 2006).

Coded portions of blog posts did not always fit neatly into only one coding category. This finding suggests the complexity of trying to adjust to and make sense of a host culture by the sojourner. Her blog posts provided evidence of this complexity as she reflected on multiple elements of intercultural interaction and adjustment within a single sentence or paragraph.

Below, the four specific research questions guiding the study are discussed. The evidence of culture shock provided in this personal travel blog was multifold, multifaceted, and congruent with many of the well-established elements of culture shock reported in the field discussed earlier in the paper. The degree of strangeness, to borrow Gudykunst and Kim (2003) term, between the Zimbabwean and U.S. American culture is relatively high. This means that the cultures are markedly different in important respects, it is therefore understandable that the blogger would experience relatively high levels of culture shock and thus those experiences would be reflected on in her blog posts.

Some posts signaled the blogger’s awareness of specific elements of culture shock and even the phenomena itself. For example: “I was jetlagged and culture shocked…” and “I woke up feeling homesick…” and “…it makes me feel depressed and a little panicky….” Other posts that were coded as culture shock, the blogger may or may not have been aware are elements of the experience of culture shock. Posts that talked about the loss of familiar cues and the disorientation or inability to make sense of familiar situations were examples of this. This evidence suggests that in travel blogs, intercultural communication researchers may find fairly rich data sets about culture shock to study.

The evidence of cross-cultural comparison in the personal travel blog was, overwhelmingly, reflections comparing host and home cultures, both in environment and cultural practices. This evidence suggests that bloggers use cross-cultural comparison in their writing as a way to make sense of their experiences in the host culture and that this is a strategy that seems to work well in the context of the quality of blogs as a medium that enables/encourages reflection. This conclusion is consistent with the literature finding that blogs are a place for reflective thinking (Chong 2010; Lee 2010; Bonk and Zhang 2006; Dos and Demir 2013; Gregg 2006).

There was also some evidence that third cultures were used as yardsticks for comparison. The blogger used her experience in the Irish and English cultures to help her make sense of her Zimbabwean experience. This suggests that travelers who travel to multiple cultures might have more fodder for cross-cultural comparison based on their experiences in those cultures and that reflection might appear in their blog posts.

Evidence of the blogger experiencing intercultural communication challenges in the blog was few and far between. It is not clear if this rather sparse reflection on this element of the blogger’s experience was due to there being limited communication challenges on her sojourn to Zimbabwe, or if the limited focus on communication challenges simply demonstrates this was not an area of reflection she chose to blog about. A third explanation might be that the blogger was not aware of some of the verbal and or nonverbal communication cues she missed or did not understand.

The evidence of intercultural adaptation was the second least reflected upon element in the blog. This is not a surprising result given that adaptation, as part of the acculturation process, takes place over an extended period of time and the blogger’s Zimbabwean sojourn was only two weeks long. Using language or terms from the host culture was the most common way the blogger demonstrated some form of adaptation attempt. This may be a form of language accommodation where communicators seek to accommodate those they are communicating with in the code they use Gudykunst and Kim (2003). This may also simply be a demonstration of the blogger ‘trying out’ newly learned vocabulary. The travel blogs of sojourners who spend longer periods of time in a culture, or who return to the same host culture multiple times might provide more evidence of intercultural adaptation than seen in this case study.

## Reflection’s role in the blog

The sojourner’s travel blog had examples of two of Freeman and Brett (2012) types of reflection discussed earlier. This paragraph from a blog post is an example of Descriptive Reflection: “I know I missed some good stories/landmarks, so hopefully they’ll [the hosts] tell me again later when I have more wits about me. I think aside from tiredness I had my introvert shields up and nothing was getting through while they talked”. The blogger explains both a purpose of the blog (to record information about the place she is visiting) and the reasons why she fears she might be failing on the first day of her trip (because she is too tired and feeling too introverted to engage with the conversation around her). The paragraph illustrates two elements of culture shock—withdrawal and not being able to fully cope in a normal situation. Because Descriptive Reflection is the most “I” centered of Freeman and Brett’s identified types of reflection, it is both the easiest to identify and the easiest for many bloggers to produce because, since these are personal interpretations, the writer can’t get it “wrong”.

A second, longer passage from the blog demonstrates Dialogic Reflection:

Again, I feel inept describing anything. I often can’t tell the difference between the road and “the mud” (it isn’t muddy here right now because it isn’t rainy season despite today’s rain). It’s all red and looks the same and none of it is really what you’d call a smooth ride. I finally had to ask if the houses we were driving past were middle class because I just can’t tell. I feel dense. I should be able to tell, but when I try to apply my American sense of how to decipher the clues, it doesn't work. At home, a house without screens or visible windows would only be seen in a poor neighborhood, for instance, so when I see this in Rick’s neighborhood, my brain gets confused. The yards all look wild to me. Rick will refer to something as “the bush” or “a farm” and I can’t see how it is different from anything else. In the Midwest, if you are out walking, you are either crazy, lost your driver’s license due to a DUI, or you are too poor for a car. Here, as I mentioned, most people walk. You don’t realize how limited your vocabulary is until you try to apply your own sense of what something is to a whole new landscape and culture.

Five different coded elements of culture shock are present in this reflection, including a loss of common cues, and a questioning of identity. In this section, the blogger makes the transition from the close, subjective view of what she observes and moves into a perspective that, while aware of her own reactions and feelings, is more consciously aware that signs an individual uses to categorize information are unreliable when put into an unfamiliar context. Though she does not yet have the data she needs to understand how to interpret what she experiences, she does demonstrate a need for that data. Once she has the needed information, it might be assumed that blog posts would move from Dialogic Reflection to an even more objective and comprehensive Critical Reflection, which would investigate multiple ways of interpreting both her experience within the larger social and cultural world of the host culture.

## Motivations for blogging

As noted earlier, bloggers have different goals or reasons for blogging (Ting et al. 2014; Whalen et al. 2013; Gregg 2006). When considering the reflections on intercultural communication found in this sojourner’s travel blog, it is useful to consider her motivations for writing during her sojourn:

“My motivations for blogging about my first trip to Zimbabwe were fourfold. It would be difficult to focus on a primary, single motivation. Those motivations were:

1. To maintain a chronological diary so I wouldn’t forget what was seen/done on any given day.
2. To compile “raw data” of both what I saw/experienced and my initial reactions to it that could be referred to later for more formal writing about the trip.
3. To share my trip with friends and family who had expressed interest in reading about it.
4. To have an audience—whether that is a blog readership or a single person to whom a letter is addressed, having the *idea* of someone receiving my words is an important daily practice, so in this way, blogging about Zimbabwe was not a conscious choice. It was simply what I do: write for a receiver other than myself”.

Consistent with the literature, the blogger had multiple motivations for writing while on her sojourn in Zimbabwe. Motivation two above can be read as most closely approximating the kind of cognitive and affective processing that needs to take place for someone in a host culture to become more interculturally aware. She clearly wants to both describe her experiences, but also wants to consider her reactions to the interactions and observations she makes in the host culture.

Also consistent with the literature and a stated motivation of this sojourner in her blogging practice is having an audience to write for Lee (2010). This idea of writing for an audience suggests that bloggers, this blogger included, would want details in their posts—having detail of interactions, the environment and reactions to those make it more likely than not that travel blog posts will include some intercultural communication reflections.

## Limitations of the study

The case study methodology limits the generalizability of the findings because only one blog was studied. Other travel blogs need to be examined for evidence of reflections about the four intercultural communication constructs before generalizable claims about the utility of travel blogs for intercultural communication research can be made. (Because of the unequal distribution of technology and Internet resources in developing nations, those developing nations might have fewer voices online to study, thus resulting in preferential voicing of members from the developed world.) Secondly, the relatively short sojourn of the blogger to Zimbabwe limited the interaction she had with Zimbabweans and her experiences in the host culture. This may have skewed the findings toward reflections on culture shock and limited reflections on intercultural adaptation.

## Conclusion

Among the benefits of studying travel blogs is that they are freely and publically available on the Internet so researchers can gain easy access to these reflections on intercultural interaction. This is important because if the writing is publically available, it allays the ethical concerns a researcher might have for analyzing interpersonal communication in intercultural settings—the blogger has provided the details for a public audience and this therefore implies that the blogger is not concerned about the confidentiality of this information. Secondly, through travel blogs researchers can gain access to studying reports of intercultural interactions that takes place in widely dispersed geographic regions that researchers may not have the resources (time and/or money) to access in person. This is important because researchers often have limited resources (both time and money) to conduct robust intercultural communication studies, and blogs allow access relatively cheaply and easily.

A related third benefit to studying travel blogs is that in an increasingly globalized world where travel to distant countries and cultures is affordable, possible and increasing, sojourners write blogs from their travels in cultures or areas of the world that are not well studied in the field (like Zimbabwe for example). Studying blogs from places that have not had much systematic intercultural communication focus in the field might enable more knowledge to be generated about intercultural interactions taking place in these understudied cultures. A fourth benefit relates to methodology; travel blogs are written reflections performed in the host culture at the time the intercultural interactions are occurring, providing potentially rich data sets that are constructed while the sojourn is taking place. Asking sojourners to self-report those interactions at a later date on instruments like a survey or in focus groups may not provide such detailed or accurate recall. Fifth, the characteristics of blogs discussed earlier in the paper suggest that the medium encourages the kind of reflection that allows for detail and specific example, providing useful data for intercultural communication researchers to analyze.

Among the limitations of using blogs to study reports of intercultural interactions is that researchers are relying on the sojourner blogger’s interpretations of the interaction alone, and not receiving data from the host culture perspective. Second, there is also the concern about some bloggers and the degree to which they “maintain a belief in the notion of author-as-authority” (Gregg 2006, p. 154), and the degree to which their travel posts might make inaccurate assertions about host cultures rather than being reflective about those experiences. Thirdly, sometimes bloggers take on the role of provocateur Gregg (2006) and this might lead to the writing of controversial posts for the sake of stoking controversy. Fourth, blogs can be transient in nature as bloggers lose interest in maintaining their blogs Laff (2007) and this could reduce the archival value of a blog set.

Despite these limitations, this pilot study has demonstrated that travel blogs provide evidence of reflections on intercultural interaction taking place in a host culture that may be used by intercultural communication researchers. Different kinds of blogs may provide other data, for example analysis of the blogs of student sojourners studying abroad may provide data about their intercultural experiences that would allow programs to provide additional programming or support for other students going through the study abroad sojourn. Another potential sojourning population that might provide data for analysis through their blogs could be international volunteers, like those in the Peace Corps. Analysis of their blogs may provide researchers a window into their sojourns that are not currently being utilized or analyzed in a systematic way. This pilot study has suggested the potential of blogs as a data source and further research may shed light on potential applications of this knowledge.
